# Management of Fruit Species in Urban Home Gardens of Argentina Atlantic Forest as an Influence for Landscape Domestication

**DOI:** 10.3389/fpls.2017.01690

**Published:** 2017-09-28

**Authors:** Violeta Furlan, María L. Pochettino, Norma I. Hilgert

**Affiliations:** ^1^Instituto de Biología Subtropical, Universidad Nacional de Misiones-Consejo Nacional de Investigaciones Científicas y Técnicas, Puerto Iguazú, Argentina; ^2^Centro de Investigaciones del Bosque Atlántico, Puerto Iguazú, Argentina; ^3^Laboratorio de Etnobotánica y Botánica Aplicada, Facultad de Ciencias Naturales y Museo, Universidad Nacional de la Plata, Consejo Nacional de Investigaciones Científicas y Técnicas, La Plata, Argentina; ^4^Facultad de Ciencias Forestales, Universidad Nacional de Misiones, Eldorado, Argentina

**Keywords:** landscape domestication, urban botanical knowledge, Frontier, periurban agriculture, Ethnobiology

## Abstract

Home gardens are considered germplasm repositories and places for experimentation, thus they are key sites for the domestication of plants. Domestication is considered a constant process that occurs along a continuum from wild to managed to domesticated populations. Management may lead to the modification of populations and in other cases to their distribution, changing population structure in a landscape. Our objective is focused on the management received in home gardens by perennial species of fruits. For this, the management practices applied to native and exotic perennial fruits species by a group of 20 women in the periurban zone of Iguazú, Argentina, were analyzed. In-depth interviews were conducted, as well as guided tours for the recognition and collection of specimens of species and ethnovarieties. Sixty-six fruit species managed in the home gardens were recorded. The predominant families are Rutaceae, Myrtaceae, and Rosaceae. The fruit species with the highest number of associated management practices are *pitanga* (*Eugenia uniflora*) and *pindó* (*Syagrus rommanzoffiana*). The 10 species with the highest management intensity are (in decreasing order of intensity) *banana* (*Musa* x *paradisiaca*), *palta* (*Persea americana*), *pitanga* (*E. uniflora*), *mango* (*Mangifera indica*), *cocú* (*Allophylus edulis*), *mamón* (*Carica papaya*), *guayaba* (*Psidium guajava*), *limón mandarina* (*Citrus* x *taitensis*), *güembé* (*Philodendron bipinnatifidum*), and *mandarina* (*Citrus reticulata*). Among the families with the greatest modifications in their distribution, abundance and presence of ethnovarieties in domestic gardens, are the native Myrtaceae and the exotic Rutaceae. The main management practices involved are cultivation, tolerance, transplant and enhancement in decreasing order. It can be concluded that in Iguazú, fruit species management shows both in plant germplasm as in environment a continuum that through tolerance, transplant and cultivation *latu sensu* has derived in a mosaic of species in different management situations, which in turn are representative of an anthropogenic landscape in constant domestication and change.

## Introduction

The interactions between nature and culture formed the landscape, represented by the dynamic relationship between physical spaces, people, and natural resources throughout history. This relationship is constantly shaped by cosmovisions, values, and perceptions as well as by the biodiversity of the environment (Balée, 1998; Brodt, 2001; Pochettino et al., 2002; Davidson-Hunt and Berkes, 2003; Berkes and Turner, 2006; Toledo and Barrera-Bassols, 2009; Capparelli et al., 2011; Ladio, 2011). The transformation of the environment based on cultural criteria leads to the creation of a specific landscape. This co-created environment becomes a way of extending the domestic unit, where management and domestication of the species are primary tools (Stampella, 2015).

Family farming in Latin America is diverse according to the high variability of cultural groups. The way in which settlers appropriate nature influences the generated agroecosystems in both plant diversity and in its management (Paulus and Schlindwein, 2001; Toledo and Barrera-Bassols, 2009). From people and plants constant relationship, located biocultural entities arise which have the capacity of transforming each other and, consequently, the inhabited landscape (Lema, 2013). In this sense, home gardens are important places for experimentation as a part of an inhabited landscape (Pochettino et al., 2012). That is why they have international recognition as key sites for species domestication and germplasm repositories (Huai et al., 2011).

Over the twentieth century, scientists tried to categorize cultural groups on the basis of the way they work the land. However, archeological evidence showed there were numerous intermediate ways of land management and strategies that do not fit into cultivation or gathering as they were understood at that time (Harris and Hillman, 1989). Thanks to that discordance, it was triggered the interest of unraveling other forms of management that could lead to the phenotypic and genotypic modification of a species. To understand these kinds of managements, Casas et al. (1996), working in Mexico, proposed a categorization of practices observed in Nahua and Mixtec groups. At the same time Clement (1999) proposed a theory regarding landscape domestication phases together with plant domestication processes for Amazonian crops.

Home gardens are structured and maintained over time by the constant implementation of management practices like tolerance, enhancement, protection, transplantation and planting of particular species or individuals (Casas et al., 1998). These practices lead to selective maintenance of wild vegetation and species of cultural importance, encouraging the emergence of phenotypic divergences settled in local preference criteria and domestication process itself (Casas et al., 1996).

The concept of perennial fruit species is used in this text according to Miller and Gross (2011) to group those plants that are grown in home gardens mainly for fruit consumption (although they have multiple uses) and are generally long live perennial. Botanically the group involves herbs (as *Musa* section), epiphytes (as *Philodendron bipinnatifidum*), palms (as *Syagrus rommanzoffiana*), shrubs and trees (as *Malpighia emarginata* and *Psidium guajava*, respectively).

Numerous studies demonstrate the process of domestication in perennial fruit species. Some well known examples belong to Cactaceae, Lauraceae, Anacardiaceae botanical families and also Amazonian species of the Annonaceae family (Miller and Schaal, 2006; Bost, 2009; Clement et al., 2010; Parra et al., 2010; Blancas et al., 2013; Aguirre-Dugua et al., 2013; Lins Neto et al., 2014).

Inside Argentina Atlantic Forest, in the province of Misiones there are four principal cities according to its economic and politic importance (Instituto Nacional de Estadísticas y Censos [INDEC], 2010). These cities are Posadas, Oberá, Eldorado, and Puerto Iguazú. The last one is surrounded by natural protected areas and is part of a green corridor called “Corredor Verde Misionero” (García Fernández, 2002), it also shows a very complex cultural composition (Belastegui, 2004; Furlan et al., 2016). For these reasons this contribution focuses only on Puerto Iguazú as a study case. The landscape in Puerto Iguazú, mostly present in periurban area, is defined as a domesticated landscape. The main characteristics of this landscape are correspondent with the intensity of domestication proposed by Clement (1999) as a cultivated area with swidden/fallow structure. Although all forms of landscape domestication are put in practice in the region, many of them occur simultaneously. Domestication process occurs with different intensity. This intensity is related to the complexity of management practices applied to the plants, the number of practices carried out and the number of people who carry them out in a particular population (González-Insuasti and Caballero, 2007). Through the recognition of management intensity, mediated by the biological characteristics of the species in question, it can be stated its cultural importance (González-Insuasti and Caballero, 2007; Blancas, 2013). Previous works by the research group (Furlan, 2017) highlighted the importance of fruit species in the domestic gardens of Puerto Iguazú. The word fruit comes from the Latin “*fruor*” that means to enjoy (Simpson and Ogorzaly, 1995). The main use given to perennial fruit species in Puerto Iguazú is associated with this perspective of enjoyment and complement to food and medicine. Most of the fruits are consumed at the same time of their maturation and without mediating too many preparations or preserves. The objective of this research is to determine which perennial fruit species are managed and the most common management practices for them in home gardens of the periurban of Iguazú.

### Study Area

#### The Atlantic Forest in Misiones Province

The Atlantic Forest is classified as one of the hot spots of biodiversity in the planet (Myers et al., 2000; Mittermeier et al., 2004), and Argentina holds the bigger continuous remnant of this biome, which covers approximately 10,000 km^2^ (Izquierdo et al., 2011). The province of Misiones is located in the southern limit of this ecorregion (Galindo-Leal and de Gusmão Câmara, 2003; Placci and Di Bitetti, 2006).

This biome is distributed for 3300 km along Brazil coast, southeast of Paraguay and northeast of Argentina. This area is characterized by a semi-deciduous forest with differentiated strata, abundance of epiphytes, bamboos and lianas (Campanello et al., 2009; Montti et al., 2011). The weather of the region is subtropical humid without dry season. The average annual rainfall is 2000 mm and the mean annual temperature is 20°C (Campanello et al., 2009). Misiones province is one of the most diversified regions of Argentina (Placci and Di Bitetti, 2006). Nowadays the Atlantic Forest of Misiones hosts 1.000.000 inhabitants and 26.500 familiar agroforestry systems (Censo Nacional Agropecuario [CNA], 2002). The interactions between people and forest has been studied from an ethnobotanical perspective (Keller, 2008; Zamudio, 2012; Kujawska and Luczaj, 2015) and from an ecological perspective (Izquierdo et al., 2008, 2011).

#### Socio-Cultural Characteristics of Misiones and Puerto Iguazú

The present research focuses on Puerto Iguazú, a city located in northwest of Misiones, bordering with Brazil and Paraguay (Nuñez, 2009). This area is known as a Triple Frontier (Rabossi, 2010). The city of Puerto Iguazú, together with Foz do Iguaçú (Brazil) and Ciudad del Este (Paraguay), create an important center of attraction for population inside the province (Barreto, 2002). Also, for Latin America the area has the biggest cities in relation of all the Triple Frontiers of the region (Rabossi, 2010).

Family agroforestry systems in Misiones are called “*chacras*,” in each one of them people make multiple use of resources (Chifarelli, 2010a). Among the activities which characterize them there are a diversity of crops, forestry production, citric production, extraction of timber and non-timber forest products (Chifarelli, 2010b).

**FIGURE 1:**
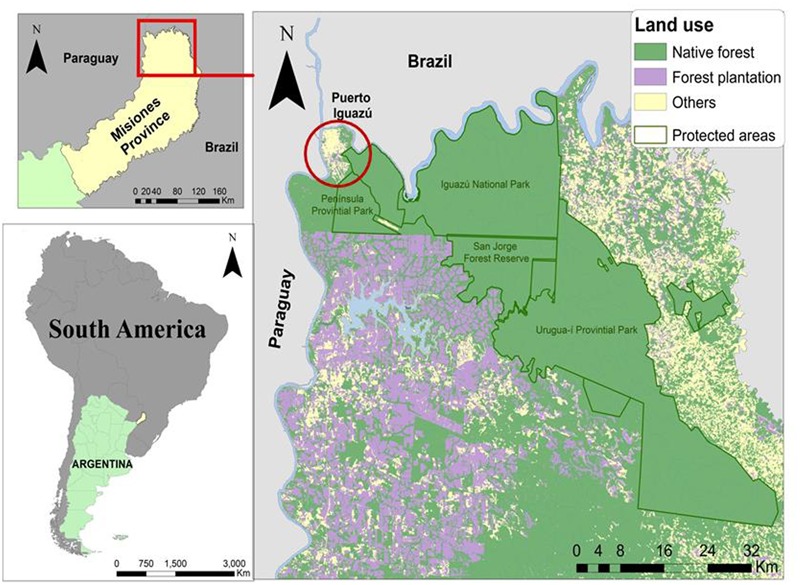
Study Area.

The most important economic activities of the region are silviculture and agriculture complemented by livestock farming (Instituto Nacional de Estadísticas y Censos [INDEC], 2010). Tourism, on the other hand, represents the main source of direct and indirect incomes for Puerto Iguazú (Nuñez, 2009). The area presents a constant migration flow from neighboring rural areas (Izquierdo et al., 2008) and an ethnic composition similar to the rest of the province, being a pluricultural context with influences of *criollos, guarani*, eastern Europe, Brazilian and Paraguayan traditions (Furlan et al., 2016) (**Figure 1**).

The productive landscape as the social scenario of Misiones is complex. The territory of the province has been occupied by Guaraní linguistic groups long before the province and even national organization. Most of these groups came from Amazonas river basin to the area (Cadogan, 1957) where they inhabited since, at least, 1200 years (Poujade, 1995). In spite of this the region was considered during the past century, as one of the under populated areas of the country. That statement lead to colonization plans that brought together people from Eastern Europe, Argentinians from other regions, Brazilians and Paraguayan migrants to the same place. Land property was different according to formal or informal colonization. Until today there are serious tensions about land tenure and property rights for most of local population (Schiavoni, 2006). Currently three languages are used in everyday life Spanish, Guaraní and Portuguese (Instituto Nacional de Estadísticas y Censos [INDEC], 2010).

According to official records, Puerto Iguazú had a population of 32,038 inhabitants and there were 7,580 dwellings (Instituto Nacional de Estadísticas y Censos [INDEC], 2001). There are fundamental relations of interdependence with neighboring cities operating for city functioning (Nuñez, 2009). Different land use planning were designed although they have not been implemented and at present the city lacks a proper planning (Cammarata and Gandolla, 2006).

The city is inhabited by a pluricultural population with diverse traditions which influence in its way of production. Settlers maintain family and work nets with neighboring cities (Izquierdo et al., 2008; Furlan et al., 2016). Conservation areas as well as Paraná and Iguazú rivers limit the expansion of the city. Land use in Puerto Iguazú is organized in areas. Downtown area is dominated by tourism industry (such as hotels and restaurants) and Periurban is dominated by agricultural activities. Nevertheless, family farming activities can be found in domestic units of both areas (Furlan, 2017).

Periurban area is understood, according to Barsky (2010), as a territorial complex of dynamic borders that includes elements of rural and urban land; it represents a transitional area which borders are dynamic and depend upon the rhythm of urbanization. The expansion of the agricultural border which took place in the last century in Misiones was structured upon spontaneous occupation (Schiavoni, 1995). Same patterns of occupation were repeated during the expansion of the urban area, which was structured upon the constant process of mobility of local people (Nuñez, 2009; Furlan, 2017). They carry those movements out along time between different territories of the Triple Frontier, in pursuit of the most favorable conditions for their families. This constant change of domesticated landscape has influenced the selection of plants managed in each domestic garden (Furlan, 2017).

Generally, women are the principal managers of home garden diversity and the products generated are for internal use of the domestic unit and occasionally sales (Furlan, 2017). Each home garden of Puerto Iguazú is formed by a variable number of microenvironments, being the main ones garden, park, orchard, *chacra* (plot area used for planting staples as cassava, maize, and beans), *monte* (native forest area in different stages of conservation) and *capuera* (area of secondary forest formerly used for annual crops as *cassava*) (Furlan et al., 2015). The detailed characteristics of these microenvironments are described in Furlan et al. (2015) and Furlan (2017). Specific information about richness and composition of medicinal species of Iguazú home gardens can be found in Furlan et al. (2016).

Home gardens in Iguazú have a variable number of species that ranges from 50 to 150. Most of the species held in the domestic unit have local varieties. That is why the total number of ethnospecies is as higher as 619 for the home gardens studied. The uses of the species reach a total a 747, being alimentary and medicinal plants the principal uses (Furlan, 2017). All the gardens visited were bigger than 450 square meters. From previous work is known that home gardens in Puerto Iguazú are present most of the times in plots bigger than that size, as well as that women are more prone to maintain a garden. Even more if women are aged between 30 and above years old. Seeds and plants of home gardens are obtained firstly by exchange with family and neighbors and in some occasions are bought to local sellers (Furlan, 2017).

## Materials and Methods

### Interviewing Methods

This paper is part of a bigger project that involved the first author’s doctoral thesis and postdoctoral fellowship. For this reason, the selection of interviewees for this contribution has been done carefully from a bigger sample (from 369 interviewees, 10% of Iguazú domestic units). For this contribution field work was made during 2014–2015 with 20 women living in the periurban area of Puerto Iguazú. The criteria for their selection were: to have wide diversity and variability of management practices in their gardens, have been established in Puerto Iguazú for at least 30 years and to be older than 30 years. All women involved were asked to participate of this research and the objectives, researcher’s participation and destiny of information shared during interviews were explained to them and written in an informed consent note^1^. The first contact with women was made during 2012 as part of a bigger project, for this contribution we had already previous bond. All species records refer to plants present in their domestic units.

Semi-structured interviews were carried out along with in depth interviews and guided tours through the home gardens. In each interview regarding to plant management was asked first which plants were classified as fruits of home gardens. Once we had the group of plants considered as fruits locally, we asked each women about if the plants were already there when they came to the *chacra*, then if they had moved them to other place of the domestic unit, also if they find a new plant growing if they let them standing or not. We asked if they make anything to increase the number of plants for each species and which were the special cares that they give to each one too. Which of the species were planted or removed in case they did not want them somewhere. Harvesting and pruning techniques were not specifically asked, those practices arose during the interviews.

### Botanical Determination of Perennials Plants and Management Categorization

Voucher specimens of managed perennial fruit species were collected on farm. Plants were identified by the authors and stored in the Herbarium of Instituto de Biología Subtropical (IBSIHerb) in Puerto Iguazú and in the Herbarium of Instituto de Botánica del Nordeste (CTES) in Corrientes, Argentina. The botanical origin of species was checked against “Flora del Conosur” of Instituto de Botánica Darwinion^2^. The scientific name of plants was verified using the Plant List^3^ and full name of plants and its botanical origin for the area are presented in Supplementary Table 1. For their categorization, the name of the species was maintained and the varieties recognized locally were taken into account.

Emphasis was placed on the management of all species, including ethno-varieties, without differentiating between those already domesticated species and those that only have management. This decision was made since management and diversification are a constant process that can occur both in domesticated species and in not domesticated ones, such as peaches in northwest of Argentina and citrus in northeast of Argentina (Stampella et al., 2013; Hilgert et al., 2014). Management practices for perennial fruit species were defined according to Casas et al. (1996) and Blancas et al. (2013) and were modified for this case study according to the concepts shown below.

Tolerance: It applies to the practice of keeping individuals during thinning (cleaning), pruning or previous managements. This term is also used for new specimens grown spontaneously in domestic units that are left for their development.

Protection: It involves actions to avoid damages caused by environmental factors (climatic factors, pathogens, herbivores) on the selected species. Or in order to prevent that small animals, either farm or wild, eat the new shoots of plants. Chemical pest control systems were not considered among the protection techniques.

Enhancement: It consists in favoring the number of individuals of a species or variety for example by eliminating competition, watering seeds, consciously dispersing seeds to increase the abundance of a particular species. The improvement of soil quality and the use of fertilizers (organic or industrial) were not considered in the enhancement.

Transplantation: It applies to those individuals who were naturally settled and moved or individuals who were tolerated and then relocated.

Sowing or planting: It refers to seed or vegetative propagation that involves establishing the species in a favorable place for its germination and growth. It also includes plants that are reared in seedlings and later transplanted. Vegetatively reproduced species are included in this group such as *pineapple, banana, güembe, strawberry, tuna* (*Opuntia*).

Removal: It refers to the elimination of individuals.

Harvesting and pruning were not proposed as management categories at the beginning, however, they were included afterward, and only in those cases referred to by the interviewees although it was not specifically asked. Harvesting was considered when people mentioned bringing fruits from the *monte* or *capuera* and also in the case of collecting from roads or between plots. Pruning finally was recorded as a particular management practice as settlers used it as a way to obtain greater fruition or flowering of a species or to maintain the architecture of the plant in the desired way.

In all cases, the management was registered only for plants, not for the microenvironments or productive spaces. Different life forms (trees, shrubs, and vines) were included as long as they were considered as perennial fruit suppliers for families.

### Data Analysis

In this contribution registered data were analyzed with descriptive tools as detailed afterward. Testimonies obtained during interviews were also incorporated as part of the ethnographic record and were examined qualitatively. **Figure 3** was made using R studio and ggplot2 package (RStudio Team, 2015).

**FIGURE 2:**
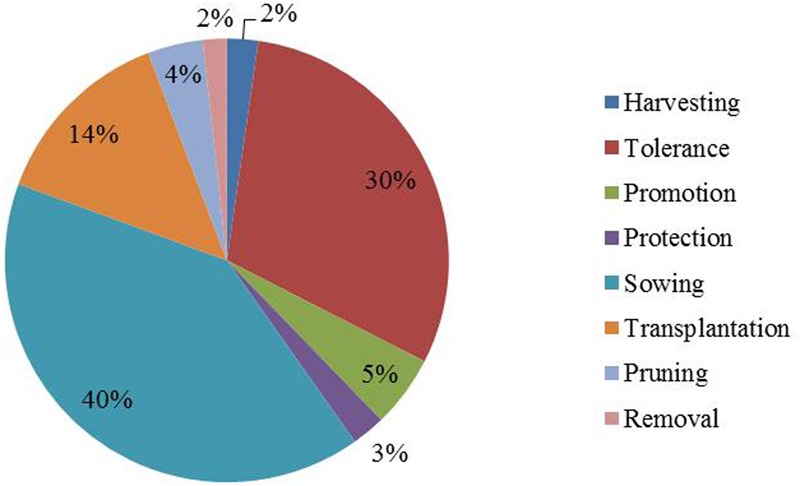
Fruits species management practices and its frequency in home gardens of Puerto Iguazú.

For quantitative data exploratory and descriptive methods were applied. In **Figure 2** percentages of management practices used for the total of domestic units of Puerto Iguazú are shown.

**FIGURE 3:**
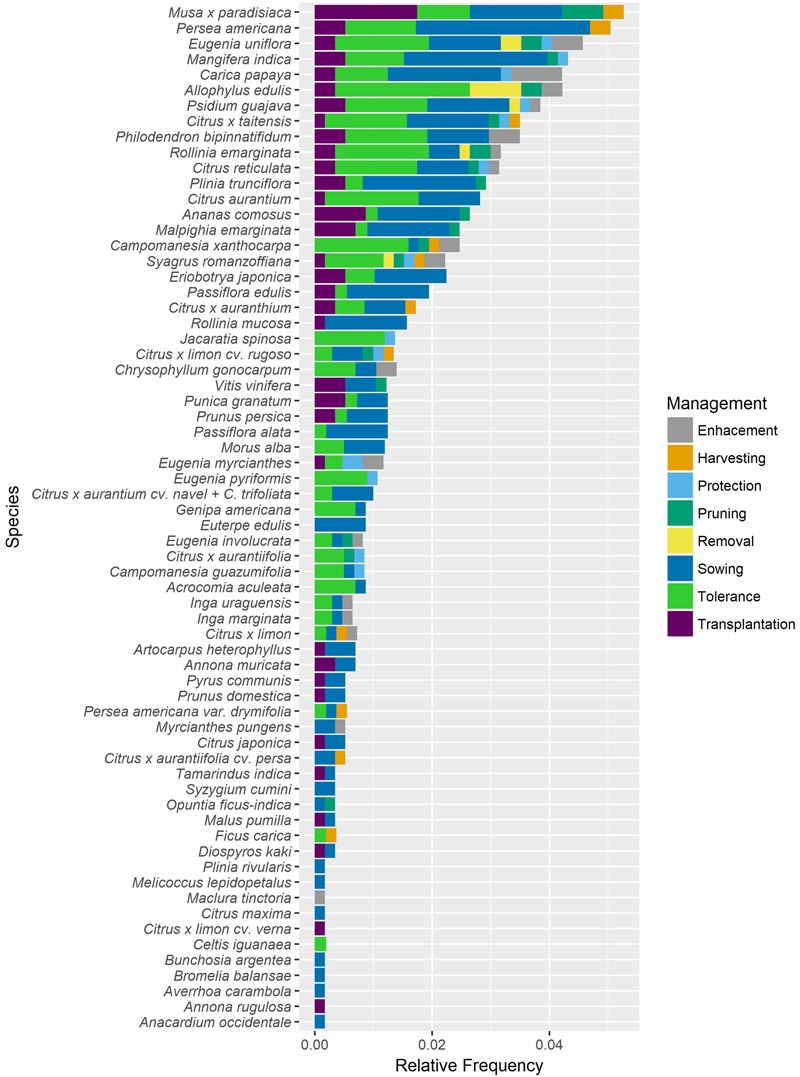
Fruit species managed in home gardens of Puerto Iguazú. Values are expressed as relative frequency. Each of the categories of management referenced is explained in the section “Materials and Methods.”

For **Figure 3** it was considered the relative frequency in which each species is managed, according to each one of the management practices. Each management practice is represented by a particular color and the length of each color bar shows the relative frequency of that practice. For example for *Eugenia uniflora* is tolerated by 9 women in a frequency of 0.016.

Simplified management intensity is calculated as the sum of all relative frequencies of practices for each species.

$$\begin{array}{c} \mathrm{Relative}frequence=\frac{\mathrm{nij}}{\mathrm{Nij}}\\ Nij=\sum \mathrm{nij} \end{array}$$

IMj = ∑ Relative frequence of practices by species
nij = number of people applying each management practice by species
i = Number of people applying each management practice
j = Each one of the species managed

For example the simplified management intensity calculated for *E. uniflora* is the sum of all relative frequencies being: Tolerance: 0.016; Enhancement: 0.005; Protection: 0.002; Sowing: 0.012; Transplantation: 0.003; Pruning: 0.003, Removal: 0.003. That is to say the management intensity is 0.045. This analysis was made to see in a wide sense if a species was having more attention. In this way when the management index throws out a bigger number is an indicator of more management attention associated to that species. If we want to know which one of the species could be interesting for future studies about domestication we can take this index into account, as a preliminary way of selecting species. After that for example González-Insuasti et al. (2008) index could be put in practice, as it is planned to do it for future research.

## Results

### General Findings

Sixty-six fruit species are managed in the domestic units of Iguazú, mostly of which are trees and shrubs. The predominant families managed are Rutaceae (12 species); Myrtaceae (11 species), and Rosaceae (6 species).

Considering the incidence of management practices according to the number of fruit species that undergo each one of them (**Figure 2**), the main management strategies are sowing (40% of the species), tolerance (30%) and transplantation (14%). On the other hand, enhancement, protection and harvesting were used for a range from 2 to 5% of the species while removal of individuals was applied to 2% of the species.

### Management Practices for Each Perennial Fruit Species

Many of the recorded species have a previous history of management, given by older interventions in the domesticated landscape. So, some of the species are object as unexpected practices, for example tolerance, while given its geographical origin, it would be more likely a sowed species. An example of this is *higo* (*Ficus carica*). In that case, tolerance -of plants previously existing in the spaces where people settled- and harvesting are the main management practices applied by the interviewees. According to natural climate and distribution of this species it is thought that specimens were sowed by someone before the new owner arrived.

The 12 fruit species with the highest number of associated management practices are (in decreasing order) *pitanga* (*E. uniflora*), *pindó* (*S. rommanzoffiana*), *mandarina* (*Citrus reticulata*), *limón mandarina* (*Citrus* x *taitensis*), *guayaba* (*Psidium guajava*), *araticú* (*Rollinia emarginata*), *cocú* (*Allophylus edulis*), *guabirá* (*Campomanesia xanthocarpa*), *mamón* (*Carica papaya*), *limón arrugado* (*Citrus* x *limon* cv. *rugoso*), *mango* (*Mangifera indica*), *banana* (*Musa* x *paradisiaca*). The number of management practices for all species can be seen in **Figure 3**.

Species dominance changes when the simplified management intensity is taken into consideration, (**Figure 3**). The 12 species with the highest simplified management intensity are (in decreasing order) *banana* (*Musa* x *paradisiaca*), *palta* (*Persea americana*), *pitanga* (*E. uniflora*), *mango* (*M. indica*), *cocú* (*A. edulis*), *mamón* (*Carica papaya*), *guayaba* (*Psidium guajava*), *limón mandarina* (*Citrus* x *taitensis*), *güembé* (*Philodendron bipinnatifidum*), *mandarina* (*Citrus reticulata*), *araticú* (*R. emarginata*), and *jabuticaba* (*Plinia trunciflora*).

The *palta* (*P. americana* var. *americana*) local varieties are selected for the preference of large and creamy fruits, while *palta anisada* (*P. americana* var. *drymifolia*) is selected because it possesses greater aroma in its leaves used to add to the “mate” (local beverage) and its fruits are not of particular importance.

All the species removed are native. These species are frequent in open areas of the Atlantic Forest and are adapted to ruderal environments; therefore they are frequently present in the domestic units that are close to forest areas, as is the case of the periurban of Iguazú. The *mamón* (*Carica papaya*) is another species that is usually removed -in particular the male stem- but did not appear in the interviewee’s mentions. Of the 17 species enhanced, only two are exotic naturalized and with great local importance such as *mandarina* and *limón arrugado* (*Citrus reticulata* and *Citrus* x *limon*, respectively). The native species that have the highest number of enhancement reports are *ubajay* (*E. myrcianthes*) and *guabirá* (*Campomanesia xanthocarpa*), while *mora de monte* (*Maclura tinctoria*) is only associated with this practice. Tolerance practice is associated to the rest of native species. Sowing practice is specially applied to *mamón, guayaba* and *guavijú* (*Myrcianthes pungens*). Protection practice is associated with several native species of the families Myrtaceae, Caricacae, and Arecaceae and with exotic species of the families Rutaceae and Anacardiaceae. From Myrtaceae family protection is applied to *E. myrcianthes* in first place and with equal frequency to *E. uniflora, E. pyriformis, Psidium guajava*, and *Campomanesia guazumifolia*. Among the species from Caricaceae family, *jacaratiá* (*Jacaratia spinosa*) is selected according to the sweetness of the fruit and is managed (through prunings and cuts) to achieve wide stems and low open plants since its stem is used to make a preserve of commercial value known as “wood marmalade”. *S. rommanzoffiana* is also protected being the only one in Arecaceae family associated to this practice. All the Rutaceae which are protected have the same relative frequency of management for that practice. While from Anacardiaceae family only *M. indica* is subject to protection.

*Celtis iguanaea*, known as *talera*, is the only species that is exclusively tolerated and does not present other recorded management practices. Sowing is the only recorded practice for the following species: *castaña de caju* (*Anacardium occidentale*), *carambola* (*Averrhoa carambola*), *caraguatá propio* (*Bromelia balansae*), *guaraná* (*Bunchosia argentea*), *pomelo* (*Citrus maxima*), *palmito* or *juçara* (*Euterpe edulis*), *ivapovó de monte* (*Melicoccus lepidopetalus*), *guaporoití* (*Plinia rivularis*) and *joaobolao* (*Syzygium cumini*). It is worth emphasizing that the fruits of *caraguatá, palmito* and *ivapovó de monte* are highly appreciated and the interviewed mentioned that it is very difficult to obtain seedlings or seeds from the forest nearby. For this reason they brought the species from other zones of Misiones province.

Among the ethnovarieties those which presented the greatest proportions of management are *mango chico* (*M. indica*), *palta con forma de pera* (*P. americana*), *palta redonda* (*P. americana*), *banana de oro* (*Musa* x *paradisiaca*), *banana petisa* (*Musa* x *paradisiaca*), *limón mandarina* (*Citrus* x *taitensis*), *limón arrugado* (*Citrus* x *limon* cv. *rugoso*).

### Some General Rules of Species Management According to the Local View

Several criteria were established that organize agricultural activities in the calendar as general rules for pruning, transplanting and sowing, for instance, temperature or moon influence. These criteria were recorded through the interviews and are frequent in the interviewee’s speech (textual phrases in Spanish, contextualized to English):

*“para podar y trasplantar hay que hacerle en los meses sin R, no importa lo que sea (la planta) sino no viene bien, se hela o se embicha* (es afectada por alguna plaga)”- pruning and transplantation must be done in months without R (in Spanish, may to august inclusive, that is winter time), no matter what (plant) it is, if not it does not grow properly, it is frozen or catches bugs (it is affected by some plague)-.

Regarding sowing it was mentioned: *“para que las plantas vengan bien siempre hay que esperar la luna (1°) de agosto, ahí cuando se siembra la mandioca, después”-*for plants to grow, you always need to wait for the moon (1st) of August, there when the cassava is planted, after-.

According to the interviewees there are many plants that can be equally “advanced in seedling or pots” so that they are ready when necessary. There are others that are planted in autumn to be cropped in winter (like certain leafy vegetables) or to “survive the frost” and then give fruit such as passion fruit (*Passiflora alata* and *P. edulis*).

Another regularity observed refers to the origin of those fruit trees that are pruned. Pruning is particularly practiced with citrus, *mango* (*M. indica*) and palta (*P. americana*) including ethnovarieties. According to the testimonies, the species of native fruit trees are not usually pruned. The main management they receive is protection, which is implemented whenever a plant grows spontaneously.

## Discussion

According to results it is important to notice that sowing is the main management practice applied in general. This result is coincident with the type of environment since it has a high level of anthropization. Generally in Latin American home gardens almost half of the species are food and half of those are fruits (Pulido et al., 2008). Martínez-Crovetto (1981) highlighted the importance of edible wild fruit trees for local people of Argentinian Atlantic Forest. From this study, it can be added that perennial fruits species are also important for urban settlers in the southwest region of the Atlantic Forest. Here, the incorporation of species from the local forest as exotic species to home gardens is a way of ensuring the provision of a variety of resources. The importance of fruits for the inhabitants of the Atlantic Forest diet was also mentioned among caiçaras by Giraldi and Hanazaki (2014) and for descendants of poles by Kujawska and Luczaj (2015). Regarding its management Keller (2008) also underlines it and its presence nearby the house in Mbya-Guarani populations and Stampella (2015) does it too for *criollos* settlers of southern Misiones. The low availability of fruit species in areas of public use and markets as well as the restriction of the use of native species in conserved areas (parks and surrounding natural protected areas) (Furlan, 2017) are likely to influence women’s motivations to incorporate these species into their home gardens in Iguazú. González-Insuasti et al. (2008) proved that land tenure is another factor that influences the decision of which species to manage with greater effort and which not. In Iguazú, land tenure is precarious for all people living in the area; the security of staying in the plot is related to the negotiating capacity that a family can have with respect to the different social actors. Therefore it is very difficult to determine the direct influence of this factor in the management of the species and its intensity in the area. However, in the new neighborhoods that are being opened could be an interesting variable to take into account for future studies. Emperaire and Eloy (2014) analyzed how the cultivation of *açaí* (*Euterpe oleracea*) in the plots of Santa Isabel do Rio Negro was considered as an “improvement” of the property and its importance for negotiation when the plot was for sale or transfer. They also pointed out that the cultivation of perennial species in the plots was a local strategy to overcome the precariousness of land tenure and achieve insertion in the urban land market. In Puerto Iguazú, the cultivation of certain perennial fruits, such as those submitted to management, can also be understood, from the perspective proposed by Emperaire and Eloy (2014), as a strategy to improve the prize of the land in case the selling is needed. Particularly it is the case of *palta, mango*, and *citrus* that are always present in the domestic units and with multiple management techniques associated. In new neighborhoods, that usually present greater land conflicts than the old ones, the new settlers are likely to choose species of rapid growth to establish in the place and, along time, to incorporate others obtaining a greater structural complexity (Furlan, 2017). This characteristic is coincident with the maintenance of perennial fruit species as shown in this text. In addition, they are of importance in the construction of the inhabited space, that is to say in the construction of the territory understood from intentionality and based in exchange relations.

The importance of perennial fruit species in the results is reflected both by the number of species managed as by the relative frequency of complex practices as sowing in the domestic units. Different species of the Myrtaceae family have been marked among the species of cultural importance for polish of the north of Misiones (Kujawska and Luczaj, 2015). In Iguazú *pitanga* is one of the species with greater intensity of management, which could also indicate a high cultural importance. This species is followed by *guava* (*Psidium guajava*), *jabuticaba* (*Plinia trunciflora*), and *siete capotes* (*Campomanesia xanthocarpa*), in contrast to the species mentioned as important at Kujawska and Luczaj (2015) which are in order of importance *S. romanzoffiana, E. uniflora, E. involucrata, Campomanesia xanthocarpa*, and *A. edulis*.

Citrus species and their varieties are largely shared with those reported by Stampella et al. (2013) for the Paraná and Uruguay basins. Stampella (2015) states that citrus in Misiones are cases of re-denomination of foreign species by local communities. The appropriation and recreation of the species and their associated knowledge are reflected in the diversity of local varieties and their uses. Citrus along with other fruit species, in Puerto Iguazú, can be included in that group. As examples, the great intensity of management of the species as *P. americana, Musa* x *paradisiaca*, and *M. indica*, and the presence of local varieties, evidenciates their importance as locally appropriated resources. At the same time those results show the principal perennial species that are part of domesticated landscape of Puerto Iguazú. The dynamism of diversification can be observed in these management practices and in their frequency.

The analysis of the number of management practices associated with a species is useful to think about which elements of the landscape are being pressured by management. To acknowledge which are those practices, their complexity and in which proportion they affect a species, allows a researcher to take into account the intensity of the species management. The simplified intensity management index applied here gives us a first clue of which of the perennial species could be interesting for pursuing future studies. This management practices can lead to frequency and distribution changes of the species and local varieties in the domesticated landscape of Iguazú home gardens as well as in the environment that contains it, the Argentinian Atlantic Forest.

Management is not the same in all individuals of the same species. This strategy is related to the search of diversification and certain logic of work by those who cultivate, which promotes the individualized management of the specimens and appreciates the intrinsic heterogeneity of the species as a value as showed in Furlan (2017). Therefore management activities particularly pruning, removal and harvesting are very variable in time and space. This means that the description of these activities and tasks are a little sample of the management universe for the species mentioned and are usually variable in the ways of carrying them out and in the times in which they are carried out in Puerto Iguazú.

Given the perennial nature of most of the fruit species managed in Puerto Iguazú, the concept of humanized biodiversity may be useful in characterizing species management. Humanized biodiversity is understood as the plants and animals that humans have altered in their biological characteristics, abundance and distribution. This concept is worked by Perales and Aguirre (2008) through several Mexican examples. For future studies, it is intended to continue using this terminology together with the analysis of management categories and their intensity as proposed at Casas et al. (1996), González-Insuasti et al. (2008) and Blancas et al. (2013). In Iguazú, as in the Andean region where Lema worked, the “*crianza*” concept (Lema, 2014) also reflects the spaces porosity and shows a mosaic of the perennial species in different management situations, which can influence in landscape domestication along time. The diachronic study of this phenomenon it is a line to continue research for future years.

Akinnifesi et al. (2010) showed how urban gardens can serve as a repository of native species and among them are several that are at risk of extinction in the original environments. Borges and Peixoto (2009) found that more than 50% of the known plants in villages within the Atlantic Forest are species from the forest. In Puerto Iguazú, in contrast to what was found by these authors, the exotic species were more frequent. It was recorded that species of Myrtaceae family in particular (almost all native) are well represented and their presence, management and local importance may be the starting point of *in situ* conservation plans of species of the Atlantic Forest. The registration of various species of the family Myrtaceae in orchards has already been described for the same phytogeographic region by Peroni et al. (2016) and their role in local conservation was also highlighted by these authors. The fruits of the Myrtaceae family are appreciated for direct consumption by local people of Iguazú. Their consumption has been also cited as of great importance for the diversification of the diet and for its nutritional contribution in other villages of the Atlantic Forest (Giraldi and Hanazaki, 2014). These species are seldom commercialized in other areas (Amaral and Guarim Neto, 2008; Kinupp and Barros, 2010) and have low availability in Iguazú local market (Furlan, 2017). Nevertheless, their availability in home gardens is not despicable, as shown in the results section. The perennial fruits species studied are also consumed by a wide variety of birds and herbivores, so they can act as a bridge between conservation areas. The fact that they are consumed by herbivores has led to hunters in Misiones to identify and use these species as a decoy to attract their prey (Giraudo and Abramson, 2000).

The use and management of fruit species is widespread in the periurban of Puerto Iguazú and management practices are similar for both native and exotic species. These results are coincident with those found by Giraldi and Hanazaki (2014) for the coastal region of the Atlantic Forest. Bonicatto et al. (2015) suggested that the practice of conservation and management of traditional seeds was extended to commercial seeds, indicating the conservation of both groups of species and varieties. In the periurban area of Puerto Iguazú, native and exotic fruit species are conserved and maintained through their management. These practices can lead to the generation of new local cultivars. This cultural selection headships to a diversification of the landscape and is intimately linked with the cultural diversity of the place (Hilgert et al., 2014), in other words to a domesticated landscape.

The Chiang Mai Declaration (WHO et al., 1988) establishes as part of conservation strategies, the propagation of native plants in agricultural systems. The study of home gardens provides relevant information to think about sustainable use and conservation of native flora, as well as understanding local ecological knowledge (Martínez, 2015). The knowledge of the phenology of the species and their management for the vigorous development of the plants put into practice by the women of Iguazú is a fundamental pillar to incorporate their look into the strategies of local conservation. The information generated on the species managed in gardens of Puerto Iguazú can serve as a substrate to think about *in situ* strategies of conservation of the Atlantic Forest of the hand of the cultivators. Regarding domestication itself it is interesting to stand out that in home gardens of Puerto Iguazú, as part of agroforestry systems, perennial fruit species are one of the principal focus of management practices. Local practices of management applied to species as well tend to the diversification of plants and landscape. Finally it reinforced the idea of urban gardens as the primary base where domestication takes place in cities.

## Ethics Statement

This study was carried out in accordance with the recommendations of ISE (2006) Ethnobiology Code of Ethics with written informed consent from all subjects. Although local law did not require this informed consent to work with dwellers in interviews is important for us to do it, that is why we always apply the international standards.

## Author Contributions

Conceptualization, data curation, formal analysis, investigation, writing of original draft and writing, review and editing was made by VF. Conceptualization, funding acquisition, methodology review and editing was made by MP and NH.

## Conflict of Interest Statement

The authors declare that the research was conducted in the absence of any commercial or financial relationships that could be construed as a potential conflict of interest.

## Footnotes

1 The consent was informed and written in all cases. Since our institution does not asses ethics on cultural studies with local population, the informed consent form was review by a specialist in bioethics. An ethics approval was not required as per institutional guidelines and national regulations, although authors decided to do field work following the International Code of Ethics in Ethnobiology (ISE, 2006).

2 http://www.darwin.edu.ar

3 http://www.theplantlist.org/
