# Supplementary material for: Management of Fruit Species in Urban Home Gardens of Argentina Atlantic Forest as an Influence for Landscape Domestication
Source: Front Plant Sci. 2017 Sep 28;8:1690. doi: 10.3389/fpls.2017.01690 (PMC5625568; doi:10.3389/fpls.2017.01690)
Supplement: Supplementary file 1 [file Table_1.DOCX]

Supplementary Material

Domestication and management of fruit species in urban home gardens of Argentina Atlantic Forest

**Violeta Furlan^*^, María Lelia Pochettino, Norma Hilgert**

*** Correspondence:** *violetafurlan@gmail.com

# Supplementary table

**Table 1**

| **Species full name** | **Botanical origin** |
| --- | --- |
| *Acrocomia aculeata* (Jacq.) Lodd.ex Mart. | Native |
| *Allophylus edulis* (A. St.-Hil., A. Juss. & Cambess.) Hieron. ex Niederl. | Native |
| *Anacardium occidentale* L. | Exotic |
| *Ananas comosus* L. (Merr) | Exotic |
| *Annona rugulosa* (Schltdl.) H. Rainer | Native |
| *Annona muricata* L. | Exotic |
| *Artocarpus heterophyllus* Lam. | Exotic |
| *Averrhoa carambola* L. | Exotic |
| *Bromelia balansae* Mez | Native |
| *Bunchosia argentea* (Jacq.) DC. | Exotic |
| *Campomanesia xanthocarpa* O. Berg. | Native |
| *Campomanesia guazumifolia* (Cambess.) O.Berg | Native |
| *Carica papaya* L. | Native |
| *Celtis iguanaea* (Jacq.) Sarg. | Native |
| *Chrysophyllum gonocarpum* (Mart. & Eichler ex Miq.) Engl. | Native |
| *Citrus* x *aurantiifolia* (Christm.) Swingle | Exotic |
| *Citrus* x *aurantiifolia* cv. *persa* | Exotic |
| *Citrus* x *aurantium* cv. *navel* + *C. trifoliata* | Exotic |
| *Citrus* x *limon* cv. *verna* | Exotic |
| *Citrus aurantium* L. | Exotic |
| *Citrus japonica* Thunb. | Exotic |
| *Citrus maxima* (Burm.) Merr. | Exotic |
| *Citrus reticulata* Blanco | Exotic |
| *Citrus* x *auranthium* L. | Exotic |
| *Citrus* x *limon* cv. *rugoso* | Exotic |
| *Citrus* x *limon* L. | Exotic |
| *Citrus* x *taitensis* Risso. | Exotic |
| *Diospyros kaki* L.f. | Exotic |
| *Eriobotrya japonica* (Thunb.) Lindl. | Exotic |
| *Eugenia involucrata* DC. | Native |
| *Eugenia myrcianthes* Nied. | Native |
| *Eugenia pyriformis* Cambess. | Native |
| *Eugenia uniflora* L. | Native |
| *Euterpe edulis* Mart. | Native |
| *Ficus carica* L. | Exotic |
| *Genipa americana* L. | Native |
| *Inga marginata* Willd. | Native |
| *Inga uraguensis* Hook. & Arn. | Native |
| *Jacaratia spinosa* (Aubl.) A. DC. | Native |
| *Maclura tinctoria* (L.) Steud. | Native |
| *Malpighia emarginata* DC. | Exotic |
| *Malus pumilla* L. | Exotic |
| *Mangifera indica* L. | Exotic |
| *Melicoccus lepidopetalus* Radlk. | Native |
| *Morus alba* L. | Exotic |
| *Musa* x*paradisiaca*L. | Exotic |
| *Myrcianthes pungens* (O. Berg) D. Legrand | Native |
| *Opuntia ficus-indica* (L.) Mill. | Exotic |
| *Passiflora alata* Curtis | Native |
| *Passiflora edulis* Sims. | Native |
| *Persea americana* Mill. | Exotic |
| *Persea americana* var. *drymifolia* (Cham. Et Schlecht) | Exotic |
| *Philodendron bipinnatifidum* Schott ex Endl. | Native |
| *Plinia rivularis* (Cambess.) Rotman | Native |
| *Plinia trunciflora* (O. Berg) Kausel | Native |
| *Prunus domestica* L. | Exotic |
| *Prunus persica* (L.) Batsch | Exotic |
| *Psidium guajava* L. | Native |
| *Punica granatum* L. | Exotic |
| *Pyrus communis* L. | Exotic |
| *Rollinia emarginata* Schltdl. | Native |
| *Rollinia mucosa* (Jacq.) Baill. | Exotic |
| *Syagrus romanzoffiana* (Cham.) Glassman | Native |
| *Syzygium cumini*  (L.) Skeels | Exotic |
| *Tamarindus indica* L. | Exotic |
| *Vitis vinifera* L. | Exotic |
